# Uric acid–associated mechanisms of coronary artery calcification in diabetic kidney disease: evidence, hypotheses, and translational perspectives

**DOI:** 10.3389/fcvm.2026.1798922

**Published:** 2026-06-11

**Authors:** Shuangqing Li, Yan Liu

**Affiliations:** Department of Nephrology, Jinhua Municipal Central Hospital (Affiliated Jinhua Hospital, School of Medicine, Zhejiang University), Jinhua, Zhejiang, China

**Keywords:** coronary artery calcification, cross-organ mechanisms, diabetic kidney disease, extracellular vesicles, multi-omics, radiomics/artificial intelligence, uric acid metabolic dysregulation

## Abstract

Coronary artery calcification (CAC) is a strong predictor of cardiovascular morbidity and mortality and progresses rapidly in patients with diabetic kidney disease (DKD). Traditional cardiovascular risk factors and mineral metabolism abnormalities do not fully explain this acceleration, suggesting the need for a broader mechanistic framework. Emerging evidence indicates that uric acid (UA) is associated with renal metabolic stress, mitochondrial dysfunction, oxidative injury, and inflammatory pathway activation in DKD. These changes may promote local renal immune activation and contribute to systemic propagation of inflammatory mediators and extracellular vesicles. In the coronary arterial wall, this environment may increase susceptibility to vascular smooth muscle cell osteogenic programming, endothelial nitric oxide imbalance, extracellular matrix remodeling, and microcalcification formation. Recent advances in single-cell sequencing, spatial transcriptomics, extracellular vesicle profiling, radiomics, and AI-based analyses provide complementary tools for identifying UA-responsive renal, immune, and vascular cell states and for generating testable hypotheses regarding CAC progression. This review proposes a hypothesis-generating UA–kidney–immune–vascular framework for understanding accelerated CAC in DKD. The framework emphasizes evidence-supported mechanisms, emerging concepts, and translational gaps, rather than establishing UA as an isolated causal determinant of CAC.

## GRAPHICAL ABSTRACT

**Figure:**
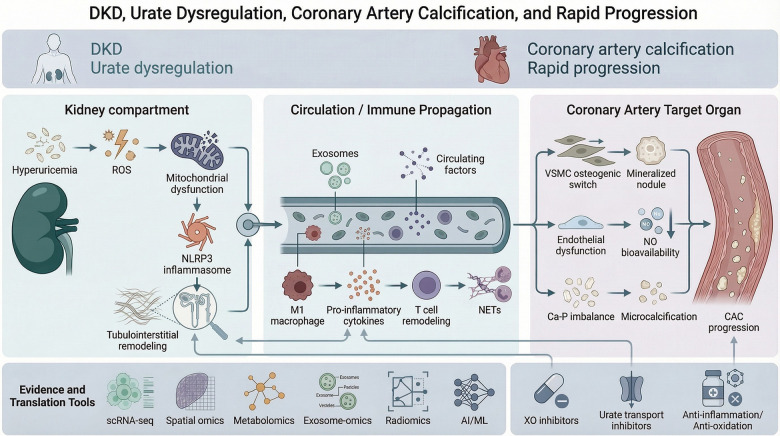
Summary framework of DKD, urate dysregulation, coronary artery calcification, and rapid CAC progression. The diagram summarizes renal metabolic stress, mitochondrial dysfunction, NLRP3 inflammasome activation, tubulointerstitial remodeling, circulating immune and EV-associated signals, and coronary vascular vulnerability. This framework presents UA-associated pathways as potential contributors to CAC progression rather than as a single established causal axis.

## 1 Introduction

Diabetic kidney disease (DKD) is a major complication of diabetes and is strongly associated with cardiovascular mortality. Cardiovascular risk increases even with mild kidney dysfunction or isolated albuminuria, suggesting that renal metabolic disturbance may impose sustained stress on distant vascular beds (1, 2). Coronary artery calcification (CAC), an indicator of atherosclerotic burden and plaque stability, occurs earlier and progresses faster in patients with DKD (3, 4). However, CAC progression may persist despite controlled mineral metabolism, indicating that mineral imbalance alone cannot fully explain accelerated calcification in CKD or DKD (5).

Across multiple cohorts with long-term follow-up and adjustment for conventional cardiovascular risk factors, this association remains robust, suggesting that kidney-, immune-, and vascular-related processes may jointly contribute to CAC progression in DKD (6). In recent years, hyperuricemia has increasingly been recognized as a key factor contributing to this gap (3). Although uric acid (UA) has traditionally been viewed as a consequence of reduced renal clearance, clinical and experimental studies suggest that elevated UA may be associated with CAC development and progression, particularly in early CKD/DKD (7, 8). Mechanistically, UA-related oxidative stress and metabolic dysfunction in renal tubular cells may activate inflammatory signaling. These signals may, in turn, affect vascular smooth muscle cell phenotype, endothelial nitric oxide homeostasis, and vascular calcification susceptibility (9, 10).

Emerging multi-omics and spatial biology studies provide additional evidence for possible kidney–immune–vascular interactions. Single-cell analyses have identified UA-associated stress signatures in renal and immune cells, while spatial omics studies suggest overlap among UA-related signaling, focal inflammation, and microvascular alterations. Extracellular vesicle (EV) studies further raise the possibility that kidney-derived signals may be transported to vascular tissues (11–13). Meanwhile, radiomics and machine learning models based on computed tomography (CT), chest radiography, and nuclear imaging have been increasingly applied to automate CAC burden quantification and cardiovascular risk prediction (14–16). However, these approaches remain largely exploratory, and their clinical utility in DKD-related CAC prediction requires further validation.

**Figure 1:**
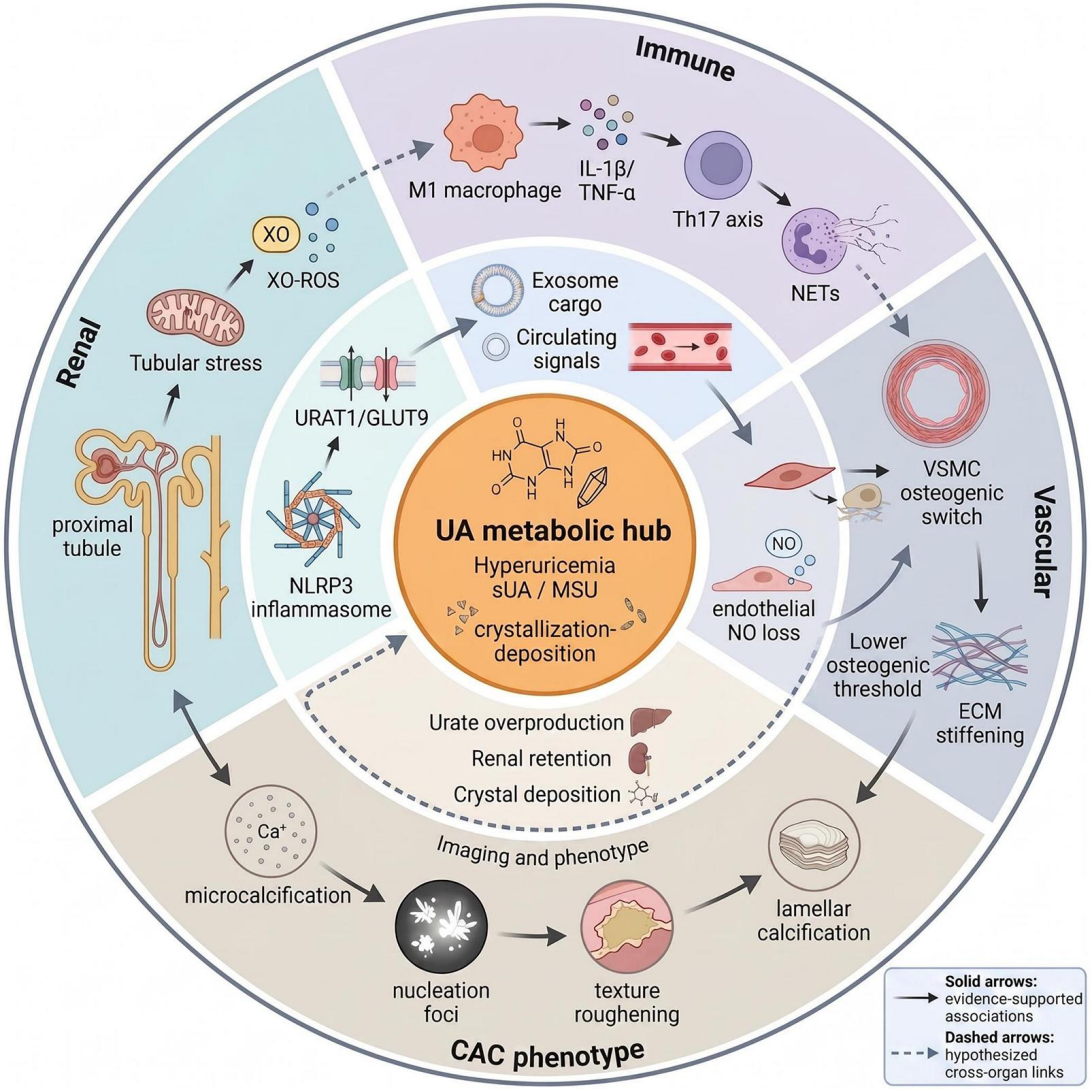
Proposed UA-associated kidney–immune–vascular axis in DKD-associated CAC. The diagram summarizes renal UA handling and XO-related UA production, renal tubular metabolic stress, inflammatory mediator release, EV-associated communication, and downstream coronary vascular vulnerability. Solid arrows indicate evidence-supported associations or experimentally demonstrated pathways, whereas dashed arrows indicate hypothesized cross-organ links requiring prospective validation. “UA metabolic hub” refers to the UA-centered metabolic node connecting tubular urate transport, endogenous UA production, XO-associated oxidative stress, and systemic inflammatory propagation.

Despite growing evidence, the role of UA-associated metabolic, inflammatory, and vascular changes in accelerated CAC among patients with DKD has not been systematically integrated. This review summarizes current evidence within a UA–kidney–immune–vascular framework. We distinguish human observational findings, preclinical mechanistic evidence, and hypothesis-generating translational concepts. Importantly, this framework should not be interpreted as proof that UA is an isolated causal driver of CAC in DKD, but rather as an evidence-organizing model to guide future research (Figure 1).

## 2 UA metabolic dysregulation in DKD and Its impact on the renal microenvironment

### 2.1 Metabolic basis of elevated UA in patients with DKD

Hyperuricemia often appears before a measurable decline in GFR during DKD progression, suggesting that altered urate handling may represent an early renal metabolic phenotype (17, 18). Hyperglycemia, inflammation, and hypoxia may affect proximal tubular urate transporters, including urate transporter 1 (URAT1) and glucose transporter 9 (GLUT9), through pathways involving nuclear factor-κB (NF-κB) and hypoxia-inducible factor-1α (HIF-1α). However, direct evidence from DKD kidney tissue remains limited, and much of the current mechanistic understanding is extrapolated from hyperuricemia or high-fructose experimental models (19, 20). Recent multi-omics and spatial transcriptomic studies in DKD models show that proximal tubular stress, inflammatory infiltration, and microvascular rarefaction can occur in spatially clustered cortical regions. These findings provide indirect support for a “reabsorption burden–inflammation amplification” concept, but this framework remains hypothesis-generating and requires further validation in human DKD tissue (12).

Enhanced tubular reabsorption alone is insufficient to account for sustained hyperuricemia (21). Increased UA production may also contribute. Studies have reported increased xanthine oxidase (XO) activity in renal tissue and circulation, which may promote purine catabolism, UA generation, and reactive oxygen species (ROS) production (22).

Overall, elevated UA in DKD may result from both enhanced tubular reabsorption and increased endogenous production. These processes appear to be linked to hyperglycemia, oxidative stress, urate transporter dysregulation, and XO-mediated purine metabolism (19, 22). In addition, DKD-related lipotoxicity can impair fatty acid oxidation and mitochondrial energy supply, thereby increasing tubular susceptibility to oxidative and proteostatic stress. Under these conditions, UA-associated oxidative injury may further reduce tubular stress tolerance and contribute to persistent mitochondrial dysfunction (23).

### 2.2 UA-associated oxidative stress and mitochondrial injury in renal tubular energy imbalance

Following the metabolic changes described above, renal tubular cells exposed to sustained hyperuricemia may develop combined oxidative and energetic stress (24). Experimental and animal studies show that elevated UA can activate nicotinamide adenine dinucleotide phosphate (NADPH) oxidase family and enhance XO-associated free radical generation, resulting in persistently increased ROS levels. Concurrently, glutathione depletion, lipid peroxidation, and reduced superoxide dismutase activity suggest impaired antioxidant defense (24, 25). Histological findings further show that oxidative stress–enriched regions often overlap with tubular degeneration, brush border injury, and interstitial collagen deposition (25).

Mitochondrial injury is another key feature of UA-associated tubular stress. Elevated UA has been linked to reduced mitochondrial membrane potential (ΔΨm), disrupted cristae architecture, and decreased adenosine triphosphate (ATP) production. Suppression of the peroxisome proliferator-activated receptor gamma coactivator-1 alpha/nuclear respiratory factor 1/mitochondrial transcription factor A (PGC-1α/NRF1/TFAM) axis may limit mitochondrial biogenesis, while impaired PTEN-induced putative kinase 1 (PINK1)/Parkin-dependent mitophagy facilitates the accumulation of damaged mitochondria (26, 27). Single-cell metabolic analyses also suggest that some tubular cells shift toward glycolysis, possibly as an adaptive response to increased metabolic stress (28). Collectively, these findings indicate that UA-associated oxidative stress and mitochondrial dysfunction may contribute to tubular energy imbalance in DKD. Rather than acting only as downstream consequences, these changes may create a vulnerable microenvironment that facilitates inflammatory activation and immune sensitization.

### 2.3 NOD-like receptor family pyrin domain-containing 3 (NLRP3) inflammasome activation as a candidate UA-associated inflammatory node

In an immune-sensitized renal microenvironment, the NLRP3 inflammasome may link metabolic stress to inflammatory activation (29, 30). Both soluble UA and monosodium urate crystals can activate NLRP3 through mechanisms such as potassium efflux, lysosomal destabilization, and ROS accumulation. Mitochondrial injury may further promote inflammasome activation through the release of mitochondrial DNA (mtDNA) and cardiolipin (31, 32). Activated NLRP3 then induces caspase-1 activation and promotes the maturation and release of interleukin-1 beta (IL-1β) and interleukin-18 (IL-18), which are important components of renal inflammation in DKD (33).

Inflammasome activation may also reshape local immune responses (29). Under hyperuricemic and NLRP3-associated inflammatory conditions, macrophages tend to shift toward a proinflammatory M1-like phenotype and release cytokines such as tumor necrosis factor-alpha (TNF-α), interleukin-6 (IL-6), and monocyte chemoattractant protein-1 (MCP-1). Tubular epithelial cell–derived chemokines may also recruit neutrophils and promote neutrophil extracellular traps (NET) formation, thereby aggravating local injury (34, 35). Transcriptomic studies further show enrichment of inflammatory response pathways across multiple immune cell populations. In hyperuricemic nephritis models, NLRP3-related inflammatory pathways have been validated in specific immune cell subsets (36). Together, these findings support NLRP3 as a candidate inflammatory node in UA-associated renal injury. However, its precise role in human DKD and its contribution to downstream vascular calcification remain to be further defined (Table 1) (25, 27, 33, 37–41).

**Table 1:** Mechanistic evidence linking hyperuricemia to renal proximal tubular injury in diabetic kidney disease through the cascade of metabolic stress, mitochondrial injury, NLRP3 activation, and structural remodeling.

Mechanistic step	Experimental or model evidence (fully documented)	Key molecules and pathways	Pathological effects
Elevated uric acid as an early metabolic burden in diabetic kidney disease (37)	Studies in patients with type 2 diabetes mellitus show that elevation of serum uric acid precedes decline in estimated glomerular filtration rate, suggesting increased proximal tubular metabolic stress and reabsorptive burden	Uric acid (UA ↑)	Enhanced early metabolic stress
Increased xanthine oxidase activity leading to concomitant elevation of uric acid and reactive oxygen species (38)	In hyperuricemic mouse models, increased xanthine oxidase activity is associated with elevated reactive oxygen species, resulting in aggravated renal oxidative stress and tissue injury	Xanthine oxidase, reactive oxygen species	Shift in purine metabolism and amplification of oxidative stress
Hyperuricemia induces proximal tubular metabolic stress and inflammation (39)	Hyperuricemia directly upregulates lactate dehydrogenase A, increases reactive oxygen species production, and promotes inflammatory cytokine release in proximal tubular cells, as demonstrated in HK-2 cell and mouse models	Lactate dehydrogenase A, reactive oxygen species	Activation of proximal tubular inflammatory responses
Hyperuricemia induces mitochondrial injury and mitophagy impairment (27)	In uric acid nephropathy models, impaired mitophagy, increased reactive oxygen species, and decreased mitochondrial membrane potential are observed, thereby promoting inflammatory responses	PINK1 Parkin pathway, reactive oxygen species	Reduced mitochondrial quality control and lowered immune activation threshold
Soluble uric acid directly activates the NLRP3 inflammasome (40)	Soluble uric acid activates NLRP3 via a reactive oxygen species-dependent pathway, leading to NLRP3 activation and subsequent interleukin-1 beta and interleukin-18 release, as demonstrated in macrophage models and validated experimentally	NLRP3, caspase-1	Maturation and release of inflammatory cytokines
NLRP3 activation in intrinsic renal cells drives diabetic kidney disease injury (33)	Podocyte-specific activation of NLRP3 aggravates proteinuria and glomerular injury, and genetic or pharmacological inhibition of NLRP3 attenuates diabetic kidney disease progression in mouse models	NLRP3, interleukin-1 beta	Local inflammation and renal structural damage
Hyperuricemia promotes renal tubular inflammation and injury progression (41)	Uric acid stimulation of renal tubular epithelial cells increases tumor necrosis factor alpha and interleukin-6 expression, leading to epithelial injury, as demonstrated in HK-2 cell models	Tumor necrosis factor alpha, interleukin-6	Tubular injury and inflammatory expansion
Hyperuricemia accelerates renal interstitial fibrosis (experimental evidence) (25)	In hyperuricemic animal models, transforming growth factor beta 1, alpha smooth muscle actin, and collagen type I alpha 1 are markedly upregulated, resulting in aggravated renal interstitial fibrosis	Transforming growth factor beta 1, alpha smooth muscle actin	Interstitial fibrosis

### 2.4 UA deposition and tubulointerstitial structural remodeling: post-inflammatory tissue-phase responses

With persistent metabolic stress and inflammation, UA deposition may contribute to the transition from inflammatory signaling to structural tissue injury (42, 43). Spatial transcriptomic and histopathological studies show that UA deposition often overlaps with tubular atrophy, interstitial inflammatory infiltration, and microvascular rarefaction. In animal models, intraluminal monosodium urate microcrystals can induce the release of damage-associated molecular patterns (DAMPs) from tubular epithelial cells, thereby stabilizing inflammatory foci and amplifying local immune responses (44–46).

As injury persists, the tubulointerstitial compartment may enter a remodeling-dominant phase (45). Reduced microvascular density can promote regional hypoxia, while fibroblast activation and collagen deposition drive renal fibrosis. Macrophage polarization toward profibrotic phenotypes may further reinforce the interaction between inflammation and fibrogenesis (47, 48).

**Figure 2:**
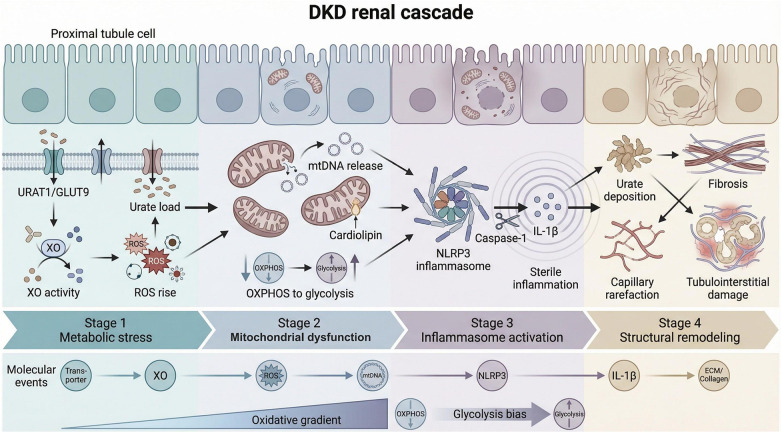
UA-associated continuous renal pathological cascade in DKD, linking metabolic dysregulation, mitochondrial dysfunction, inflammasome activation, and structural remodeling. The diagram illustrates how increased urate load, XO-related oxidative stress, mitochondrial damage, mtDNA/cardiolipin release, NLRP3 inflammasome activation, and IL-1β-related sterile inflammation may be associated with urate deposition, capillary rarefaction, fibrosis, and tubulointerstitial damage.

Overall, these findings suggest that DKD kidneys may progress from potentially reversible metabolic and inflammatory changes to a self-reinforcing cycle of inflammation, hypoxia, and fibrosis. This local pathogenic loop may provide a structural basis for renal function decline and a potential source of kidney-derived immune signal spillover, which is discussed in Section 3 (Figure 2).

## 3 From the kidney to the system: UA-associated immune propagation and vascular vulnerability

Section 2 discussed local renal oxidative stress, mitochondrial injury, and inflammasome activation. This section focuses on how renal inflammatory signals may extend beyond the kidney and contribute to systemic immune activation. For clarity, this process is organized into four candidate levels: macrophage-associated initiation, cytokine dissemination, adaptive immune remodeling, and NET-associated amplification. This framework is intended to reduce repetition of local renal mechanisms and to highlight possible links between renal injury and vascular vulnerability.

### 3.1 Macrophage-associated initiation of systemic immune propagation

Macrophages may act as an early hub of immune propagation. Hyperuricemia, hyperglycemia, and oxidative stress are associated with M1-like inflammatory polarization and glycolysis-dominant metabolic remodeling. Experimental and translational studies suggest that renal-derived inflammatory cues may promote macrophage activation and cytokine release, thereby linking local renal stress to broader immune responses in DKD (49, 50). UA-related inflammatory conditions may also activate ROS–NF-κB- and HIF-1α-related pathways, although this evidence is mainly preclinical and should not be interpreted as direct proof of causality in human DKD-associated CAC (51). Therefore, macrophage M1-like polarization is best viewed as a candidate initiating layer of immune propagation rather than an established causal driver of CAC progression (Table 2) (52–58).

**Table 2:** Experimental evidence matrix showing hyperuricemia-associated immune and metabolic reprogramming and its potential contribution to vascular lesion propagation.

Mechanistic level	Established effects of uric acid (experimental or human evidence)	Mechanistic links to vascular pathology	Evidence status
Macrophage M1 polarization (initiation)	Uric acid promotes macrophage M1 polarization through the ROS-NLRP3 pathway, leading to increased release of interleukin-1 beta and tumor necrosis factor alpha	M1-type macrophages are associated with vascular stiffening and inflammation activation	Demonstrated (cell and animal models)
Enhanced glycolysis in macrophages (metabolic reprogramming) (52)	Uric acid induces a metabolic shift in macrophages characterized by suppressed oxidative phosphorylation and enhanced glycolysis, accompanied by AMP-activated protein kinase to mechanistic target of rapamycin signaling modulation	Glycolytic macrophages promote vascular inflammation and vascular smooth muscle cell activation	Demonstrated (*in vitro*) with indirect evidence
Increased macrophage migration and infiltration (53)	Uric acid enhances macrophage migratory capacity and inflammatory activation	Enhanced macrophage infiltration is associated with local vascular inflammatory lesion formation	Demonstrated (*in vitro*)
Systemic elevation of interleukin-6, tumor necrosis factor alpha, and interleukin-1 beta (diffusion) (54)	In hyperuricemic populations, circulating interleukin-6 and tumor necrosis factor alpha levels are significantly elevated	Interleukin-6 is associated with increased vascular stiffness and elevated calcification risk	Demonstrated (human cohorts)
NLRP3-interleukin-1 beta axis activation (inflammatory amplification) (55)	Uric acid or soluble uric acid activates the NLRP3 inflammasome, resulting in increased interleukin-1 beta and interleukin-18 production	Interleukin-1 beta directly promotes osteogenic gene expression in vascular smooth muscle cells	Demonstrated (cell models)
Coagulation and endothelial activation (54)	Uric acid upregulates endothelial adhesion molecules and proinflammatory markers	Endothelial activation serves as an initiating step increasing susceptibility to vascular calcification	Demonstrated (cell models)
Th17 activation and increased interleukin-17 production (adaptive immune amplification) (56)	Uric acid promotes Th17 differentiation and increases interleukin-17 levels	Interleukin-17 promotes osteogenic differentiation of vascular smooth muscle cells	Demonstrated (animal and *in vitro* studies)
Interleukin-17-mediated vascular calcification (direct evidence) (57)	—	Interleukin-17A promotes vascular smooth muscle cell osteogenic differentiation with upregulation of Runx2 and increased calcification	Demonstrated (*in vitro*)
Neutrophil extracellular traps as a terminal amplification mechanism (58)	Uric acid promotes neutrophil extracellular trap formation through a reactive oxygen species- and peptidyl arginine deiminase 4-dependent pathway	Neutrophil extracellular traps activate osteogenic signaling in vascular smooth muscle cells and promote calcification	Demonstrated (*in vitro* and animal studies)

### 3.2 Cytokine dissemination as a candidate systemic signal layer

After macrophage activation, circulating cytokines may provide a systemic signal layer connecting renal inflammation with vascular responses. In DKD and hyperuricemia-related inflammatory settings, IL-1β, TNF-α, and IL-6 are frequently reported as representative mediators of systemic immune activation (59, 60). These cytokines have been linked to increased VSMC sensitivity to osteogenic stimuli, endothelial barrier dysfunction, and dysregulated vascular inflammatory responses (61). However, current evidence mainly supports biological plausibility and association. Direct longitudinal evidence linking renal cytokine spillover to CAC progression in DKD remains limited.

### 3.3 Adaptive immune remodeling and the Th17/IL-17 axis

Adaptive immune remodeling may provide a second layer of immune amplification. Immunometabolic studies suggest that inflammatory environments can shift T cells toward glycolysis-dependent activation, and UA-associated inflammatory models have reported enhanced T-cell activation with mitochondrial stress (62). Among adaptive immune pathways, the T helper 17 (Th17)/IL-17 axis is particularly relevant because hyperuricemic conditions have been linked to increased IL-17 activity and impaired Treg homeostasis (63).

In vascular calcification-related settings, IL-17A has been linked to osteogenic gene expression and a pro-mineralizing vascular microenvironment (64). Thus, the Th17/IL-17 axis may act as a candidate adaptive immune amplifier, although its direct contribution to UA-associated CAC progression in DKD still requires prospective validation.

### 3.4 NET-associated terminal amplification of immune propagation

NETs may represent a terminal amplification layer linking systemic inflammation to vascular injury and calcification-prone remodeling. Elevated UA and monosodium urate crystals have been reported to enhance NET formation in experimental or inflammatory settings. Circulating NET-related markers have also been associated with systemic inflammatory burden and CKD-associated vascular calcification (65, 66).

From a vascular perspective, NET accumulation has been linked to endothelial injury, increased VSMC osteogenic sensitivity, and expansion of calcified lesions in animal models (67). Therefore, NETs should be described as potential amplifiers rather than confirmed causal mediators of CAC progression in DKD.

**Figure 3:**
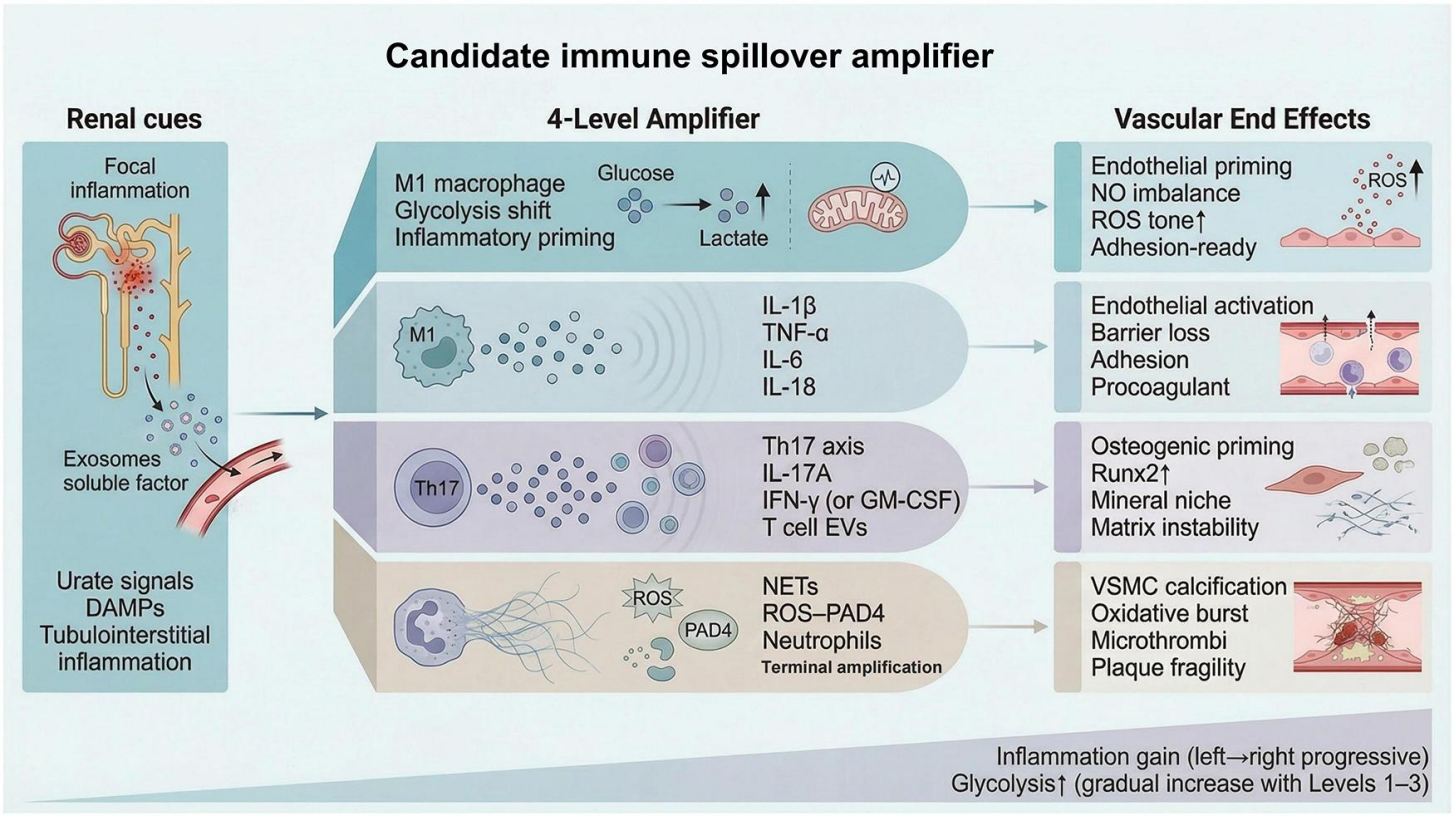
Four-level candidate immune spillover amplifier linking renal inflammatory cues to vascular vulnerability. The diagram summarizes macrophage-associated initiation, cytokine dissemination, Th17/IL-17-related adaptive immune amplification, and NET-associated terminal amplification. These pathways are presented as a biologically plausible immune propagation framework rather than a confirmed causal sequence for CAC progression in DKD.

Overall, UA-associated immune propagation can be summarized as a hierarchical but hypothesis-generating framework involving macrophage-associated initiation, cytokine dissemination, adaptive immune remodeling, and NET-associated amplification (Figure 3).

## 4 Target-organ vulnerabilization of the coronary arteries: local vascular pathways of UA action

**Figure 4:**
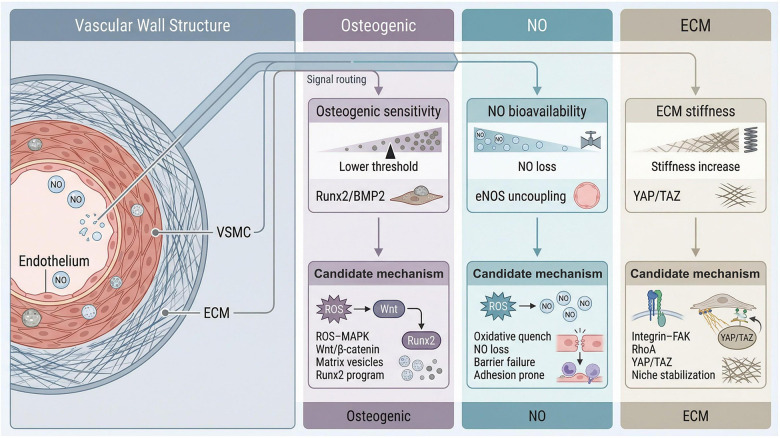
Three-axis vulnerabilization model of the coronary arterial wall in UA-associated CAC. The figure summarizes three local vascular modules: increased osteogenic sensitivity of VSMCs, reduced NO bioavailability with endothelial dysfunction, and ECM stiffness-associated mechanosensing. These axes provide candidate local mechanisms through which systemic inflammatory and metabolic signals may contribute to CAC progression.

Following the systemic immune and metabolic signal spillover described in Section 3, the coronary arterial wall may act as a local target organ where upstream cues are amplified. In this review, vascular “vulnerabilization” refers to a calcification-prone state involving three interrelated changes: lowered osteogenic activation threshold of vascular smooth muscle cells (VSMCs), impaired endothelial nitric oxide (NO)-dependent protection, and extracellular matrix (ECM) remodeling that increases stiffness and mechanotransduction sensitivity. These changes may facilitate the local translation of systemic inflammatory and metabolic stress into CAC progression (68–70). Accordingly, this section focuses on VSMC osteogenic programming, endothelial-NO axis dysfunction, and possible interactions between UA and calcium/phosphate dysregulation (Figure 4).

### 4.1 UA-associated osteogenic programming of VSMCs

VSMCs are major effector cells in vascular calcification and CAC progression (68, 71). Under stimuli such as elevated phosphate or calcium, inflammation, and oxidative stress, VSMCs can shift from a contractile phenotype toward a synthetic or osteogenic-like phenotype. This transition involves Wnt, TGF-β, and MAPK signaling, as well as matrix vesicle release that supports mineral deposition (68, 71, 72). Human plaque studies also show spatial overlap between osteogenic-like VSMCs and calcified lesions, supporting their role in CAC development (73).

Preclinical studies suggest that elevated UA may promote VSMC osteogenic programming. In animal and cell models, UA upregulates osteogenic factors such as Runx2 through ROS-MAPK and Wnt3a/β-catenin signaling. XO inhibition or reduction of UA burden has been associated with reduced aortic calcification and lower osteogenic gene expression, suggesting that UA may act as a signal amplifier in vascular calcification (74). However, direct human evidence linking UA-driven VSMC reprogramming to CAC progression in DKD remains limited.

UA may also influence vascular stiffness and ECM remodeling. Hyperuricemia has been associated with increased arterial stiffness and altered ECM architecture. In mechanobiology studies, increased matrix stiffness can enhance YAP/TAZ-mediated mechanotransduction and osteogenic programming (70, 75). Emerging evidence suggests that UA may disrupt peroxidasin-mediated collagen IV crosslinking, potentially altering basement membrane structure. Together with diabetes- or hyperphosphatemia-related ECM stiffness, this environment may activate the integrin-FAK-RhoA-YAP/TAZ axis and promote Runx2/osterix-driven osteogenic programs (76). Increased ECM stiffness may also provide stable sites for matrix vesicle–mediated calcium-phosphate deposition (70, 72). These findings support a possible biochemical–mechanical interaction, but this concept remains largely preclinical (77).

### 4.2 UA-associated endothelial dysfunction and imbalance of the NO pathway

The endothelium provides an important anticalcific barrier by maintaining NO production, limiting leukocyte adhesion, and restraining abnormal VSMC proliferation (78). When NO bioavailability declines or endothelial injury develops, the vascular wall becomes more susceptible to inflammatory and osteogenic signals (79).

Elevated UA may contribute to endothelial dysfunction through oxidative stress. UA-related activation of XO and NADPH oxidase increases ROS production, which accelerates NO scavenging and reduces NO bioavailability. eNOS uncoupling may further shift eNOS from NO generation toward superoxide production, worsening endothelial-NO axis imbalance (80, 81). *In vitro* studies show that endothelial cells exposed to elevated UA exhibit reduced NO synthesis, increased adhesion molecule expression, and impaired vasodilatory responses. In DKD-related settings, hyperglycemia, lipotoxicity, and uremic toxins may further promote NO depletion and inflammatory adhesion (10). Animal studies report that urate-lowering interventions are associated with improved endothelium-dependent vasodilation and fewer early calcified lesions (74). Thus, UA-associated NO-axis imbalance may be one local pathway through which the coronary arterial wall becomes more vulnerable to calcification-prone remodeling (80). Nevertheless, its direct contribution to CAC progression in human DKD requires further validation.

### 4.3 Synergistic calcification mechanisms involving UA and calcium-phosphate dysregulation

In CKD and DKD, CAC may continue to progress even when serum calcium and phosphate are within recommended ranges, suggesting that mineral load alone does not fully explain accelerated calcification (82). UA may contribute by lowering the osteogenic activation threshold of VSMCs. Through upregulation of Runx2, BMP2, and related osteogenic pathways, elevated UA may make VSMCs more responsive to phosphate and calcium stimulation (74).

Some investigators have proposed that UA may “left-shift” the VSMC response to calcium-phosphate stress, allowing mineralization to occur under relatively lower mineral loads. However, direct experimental validation of UA–calcium/phosphate synergy remains limited, and standardized models are lacking. Monosodium urate crystals may also serve as heterogeneous nucleation centers for calcium salt crystallization and activate NLRP3-related inflammation, offering a possible link between crystalline inflammation and mineral deposition (31, 40). Cohort studies further show that serum UA levels remain associated with CAC presence or progression after adjustment for traditional cardiovascular risk factors and selected mineral metabolism markers (6).

Overall, vascular target-organ vulnerabilization may involve three local pathways: VSMC osteogenic programming with increased stiffness sensitivity, endothelial NO-axis dysfunction, and potential interaction with calcium/phosphate-driven mineral deposition. Together, these mechanisms provide a vascular-level framework for understanding how UA-associated systemic signals may contribute to CAC progression in DKD. However, several components of this model remain hypothesis-generating and require direct validation in human studies (Table 3) (6, 83–86).

**Table 3:** Key mechanisms and evidence supporting the three-axis model of hyperuricemia-associated coronary artery vulnerability and calcification.

Local pathway module	Uric acid–related local effects	Key molecules and signals	Model type	Vascular calcification outcome
Downregulation of the osteogenic threshold in vascular smooth muscle cells (osteogenic priming) (83)	Hyperuricemia increases reactive oxygen species and reduces nitric oxide bioavailability, creating a pre-osteogenic metabolic environment	Reactive oxygen species ↑, nitric oxide ↓	*In vitro*: rat aortic vascular smooth muscle cells	Reduced osteogenic activation threshold
Downregulation of the osteogenic threshold in vascular smooth muscle cells in the cardiovascular context (84)	Hyperuricemia upregulates Runx2 and BMP2, directly promoting entry of cardiovascular cells into the osteogenic program	ERK1/2, Runx2, BMP2	*In vitro*: human coronary artery smooth muscle cells	Increased calcium deposition
Rightward shift of extracellular matrix stiffness and enhanced mechanosensitivity	Increased extracellular matrix stiffness enhances vascular smooth muscle cell susceptibility, consistent with hyperuricemia-associated extracellular matrix remodeling	YAP and TEA domain transcription factors, Runx2	*In vitro*: tunable hydrogel systems	Markedly enhanced calcification
Enhancement of endothelial inflammatory barrier signaling (local inflammatory signaling) (85)	Hyperuricemia activates NADPH oxidase, promotes endothelial inflammation, and provides a pro-inflammatory niche for vascular smooth muscle cells	NOX2, ICAM-1	Endothelial cells, *in vitro*	Increased endothelial inflammation and permeability
Uric acid crystals as a calcification nucleation center (calcium and phosphate cooperation) (86)	Monosodium urate crystals directly induce mesenchymal stem cell osteogenic differentiation and mineralization	Monosodium urate crystals, alkaline phosphatase, Runx2	*In vitro*: mesenchymal stem cells and osteoblasts	Formation of calcification nuclei
Evidence of aortic calcification in the general population	Serum uric acid levels are independently associated with increased aortic arch calcification	sUA	NHANES, cross-sectional	Aortic arch calcification and severe aortic arch calcification ↑
Coronary artery calcification in patients with chronic kidney disease (6)	After adjustment for serum calcium and phosphate levels, serum uric acid remains independently associated with coronary artery calcification scores greater than zero	sUA	Chronic kidney disease cohort	Coronary artery calcification ↑
Metabolic burden synergy between uric acid and atherosclerotic calcification (UHR and AAC)	An increased uric acid to HDL cholesterol ratio is associated with higher aortic arch calcification risk, with partial mediation by diabetes mellitus	UHR, DM	General population	Aortic arch calcification ↑

## 5 Omics and EVs: mapping candidate components of the UA-kidney-immune-vascular communication network

**Figure 5:**
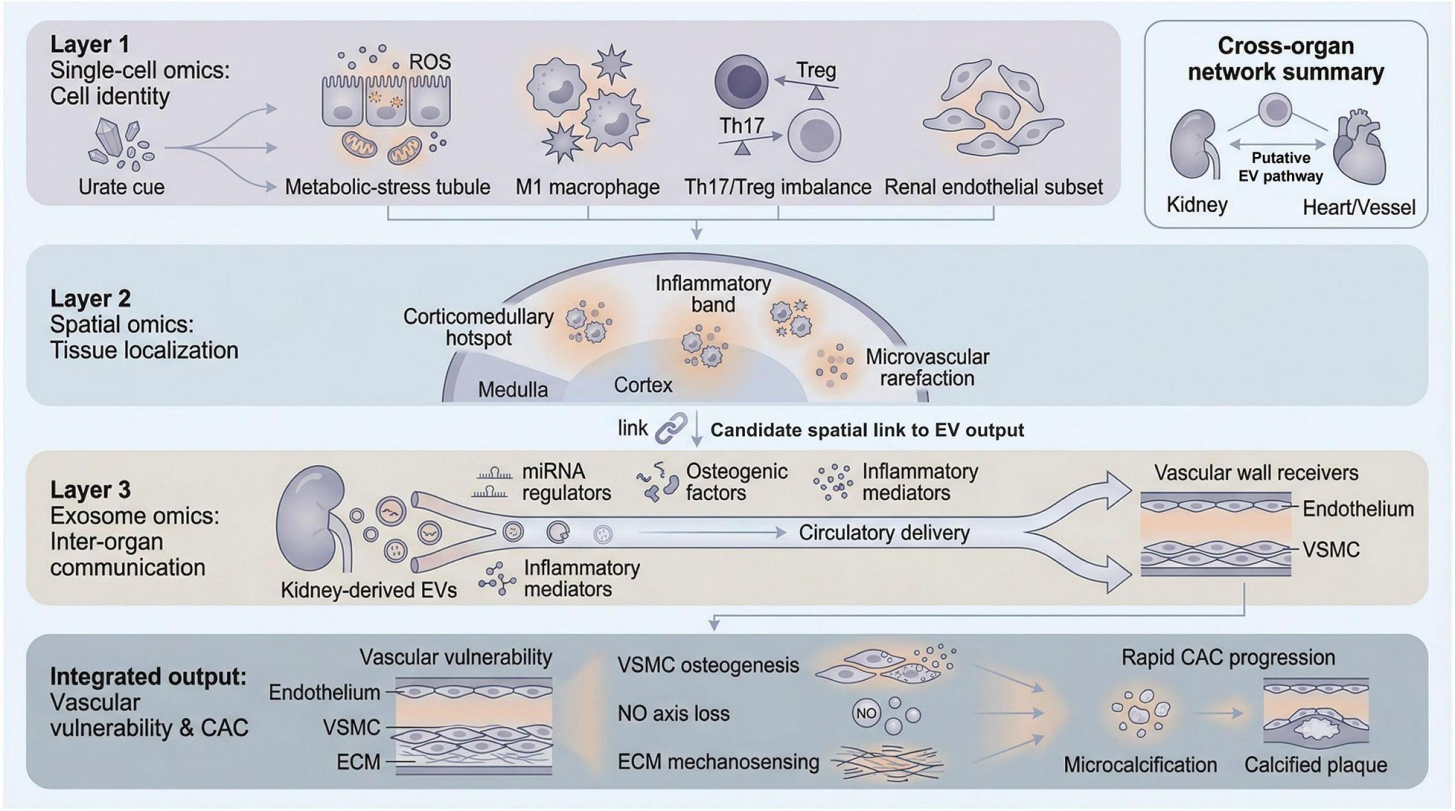
Omics-based mapping of candidate components in the UA–kidney–immune–vascular communication network. Single-cell omics identifies UA-associated vulnerable cell states, spatial omics localizes renal injury hotspots, and EV-based analyses provide candidate carriers of kidney–vascular communication. The integrated output highlights vascular vulnerability, VSMC osteogenesis, NO-axis loss, ECM mechanosensing, and rapid CAC progression as candidate downstream phenotypes.

This section summarizes omics-related evidence from three perspectives: UA-associated vulnerable cell states, spatial organization of renal injury, and EV-mediated kidney–vascular communication. Single-cell omics can identify stress-responsive cell populations, spatial omics can localize injury hotspots, and EV studies may reveal candidate carriers of systemic signals. These approaches should be viewed as complementary evidence layers rather than direct proof of a causal UA–kidney–immune–vascular axis (Figure 5).

### 5.1 Single-cell omics: identification of UA-responsive vulnerable cell lineages

Single-cell RNA sequencing (scRNA-seq) studies under hyperuricemic or kidney injury-related conditions reveal heterogeneous stress responses across renal and immune cell populations. Proximal tubular cells often show oxidative stress, inflammatory signaling, and altered purine metabolism, while renal endothelial cells may show reduced gene modules related to nitric oxide production and barrier maintenance (11, 87). In the immune compartment, infiltrating macrophages frequently display proinflammatory transcriptional programs, supporting their possible role in linking renal stress with systemic immune activation (88).

Multiome and CITE-seq studies further suggest that transcriptional or epigenetic activation may not always match conventional surface-marker profiles, highlighting the value of integrated profiling for identifying functionally vulnerable cell states (89, 90). Overall, single-cell and multiome data are useful for prioritizing candidate cell populations and pathways, but they do not independently establish cross-organ causality.

### 5.2 Spatial omics: localization of renal injury hotspots

Spatial omics adds anatomical context by showing where metabolic, inflammatory, and fibrotic signals are concentrated. In kidney injury and hyperuricemia-related models, spatial transcriptomic analyses identify proximal tubules and the corticomedullary junction as recurrent hotspots of inflammatory and profibrotic activity (91). These regions often show interstitial expansion, immune cell aggregation, and microvascular alterations, suggesting that renal stress may be organized into focal injury niches (92).

Together with evidence that renal EVs may participate in fibrosis and vascular remodeling, these spatial maps provide a possible structural basis for systemic signal release, especially near microvascular-rich regions (93). However, spatial omics mainly localizes candidate injury sites and does not prove kidney-to-coronary signal transmission.

### 5.3 Exosome omics: candidate carriers of kidney-vascular communication

EV-based studies provide a potential mechanism by which renal or immune stress signals may enter the circulation. In hyperuricemia-associated conditions, changes in circulating exosomal microRNAs and proteins, including miR-27a-5p, miR-139-3p, DPP4, and SERPIND1, have been reported. These findings suggest that UA-related metabolic states may be linked to systemic inflammatory and coagulation-related signaling (94).

Experimental studies further suggest that exosomes can promote osteogenic-like changes in VSMCs, particularly under phosphate-loaded or inflammatory conditions (95). However, prospective cohort evidence directly linking UA-associated exosomal cargo changes to longitudinal CAC progression is still lacking.

Current evidence supports EVs as candidate biomarkers and possible mediators of kidney–vascular communication. Nevertheless, their stability, tissue source specificity, and causal relevance require validation in large multicenter cohorts (96). In particular, direct lineage tracing from UA-stressed renal cells to coronary vascular cells has not been established in human DKD. Therefore, EV-mediated kidney-to-vascular communication should be considered a testable mechanistic hypothesis rather than a confirmed causal route.

## 6. AI and machine learning: hypothesis-generating tools for risk prediction and mechanistic exploration of UA-associated CAC

AI-based imaging and multi-omics models may help organize heterogeneous clinical, imaging, and molecular data in UA-associated CAC. Their main value is to identify candidate risk patterns, prioritize mechanisms, and support future trial design. At present, however, these tools should be viewed as hypothesis-generating rather than clinically validated decision systems for diagnosing or treating UA-associated CAC in DKD.

### 6.1 AI-based identification of qualitative features of CAC

Beyond the Agatston score, CT-based radiomics and deep learning methods can quantify plaque texture, density distribution, and edge morphology as imaging features of CAC heterogeneity (97). In hyperuricemia and DKD, these features may help describe high-risk or biologically active calcification phenotypes, but they should not be interpreted as direct molecular readouts (3).

Radiomic features such as texture roughness, low-attenuation regions, and edge sharpness may reflect inflammation, osteogenic remodeling, or microcalcification dynamics. However, these links remain inferential and require biological validation (97). Before multicenter use, radiomic models also require standardized feature extraction, intensity normalization, spatial resampling, batch-effect correction, and reproducibility assessment, including IBSI-based procedures where applicable (98).

### 6.2 Predictive models for UA-associated rapid CAC progression

No mature predictive model has yet been specifically developed and prospectively validated for UA-associated rapid CAC progression in DKD. Existing CAC and CCTA machine learning studies show that imaging features and clinical variables can be integrated to identify high-risk phenotypes, but these models were not designed for UA-centered mechanisms (99). Epidemiological evidence linking serum UA with CAC progression supports its potential role as a candidate variable in future multimodal models (3). Future Urate-CAC-type models should therefore be framed as transferable and hypothesis-generating frameworks, not near-term clinical tools. Model evaluation should include discrimination, calibration, decision curve analysis, and external validation to determine whether UA-related features add predictive value beyond conventional risk factors (100).

At this stage, their main value lies in hypothesis generation, feature prioritization, and trial design support.

### 6.3 AI × multi-omics integration for candidate mechanism prioritization

AI × multi-omics integration may help connect imaging phenotypes with candidate biological pathways, but its current role remains exploratory. Graph neural networks, feature selection methods, and biological network modeling have been used to identify pathway nodes and candidate targets in multi-omics studies (101). Counterfactual simulation and causal inference frameworks may further support in silico prioritization before prospective validation (102, 103).

**Figure 6:**
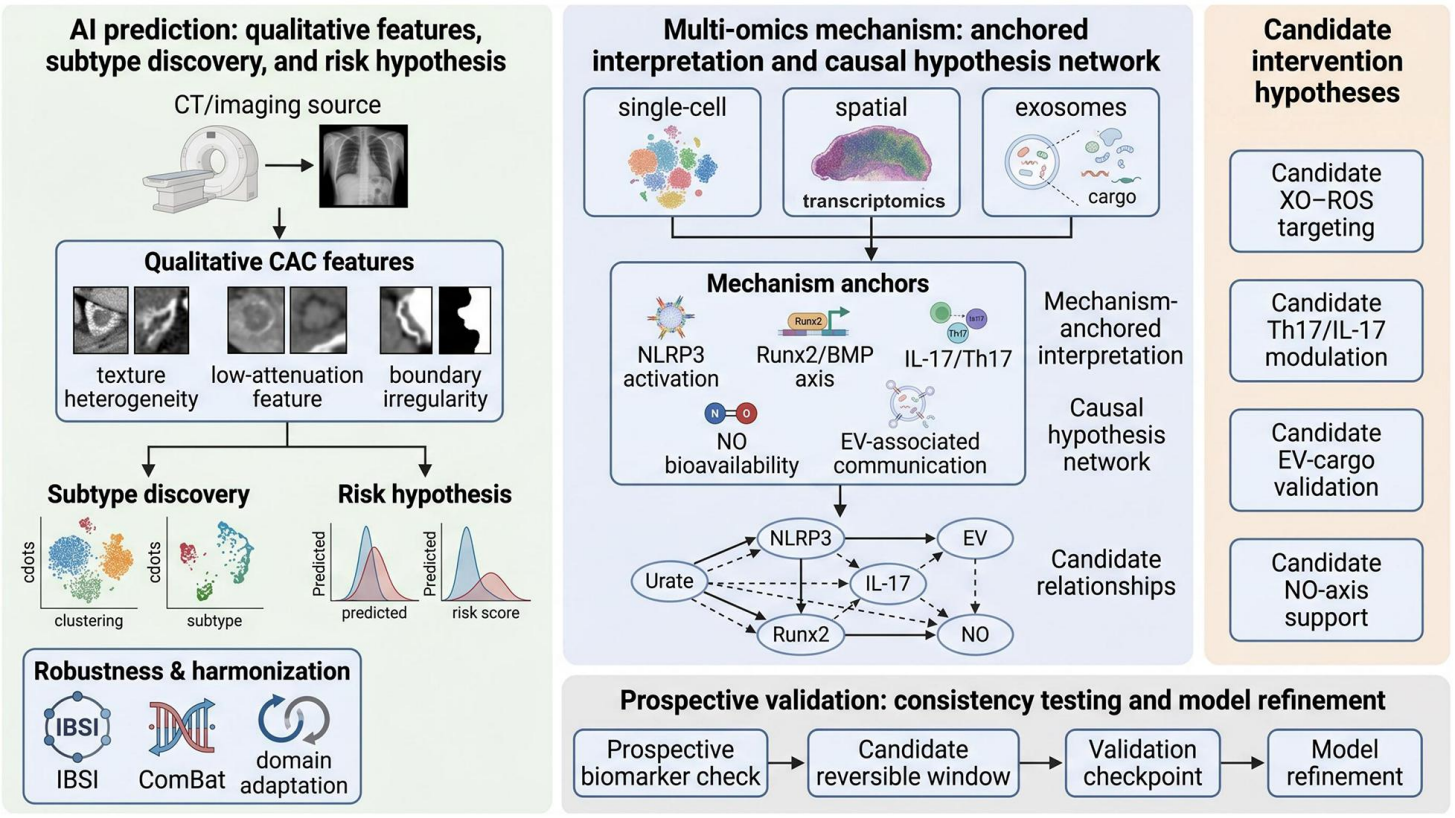
Hypothesis-generating AI–multi-omics framework for mechanistic mapping and candidate intervention prioritization in UA-associated CAC. AI-derived qualitative CAC features may be integrated with single-cell, spatial, and EV-related evidence to generate mechanism-anchored hypotheses. Candidate pathways, including XO–ROS signaling, NLRP3 activation, Runx2/BMP signaling, Th17/IL-17 activation, EV-associated communication, and NO-axis dysfunction, are shown as examples for future validation. The proposed framework is intended for mechanism prioritization and prospective consistency testing rather than for immediate clinical decision-making.

In UA-associated CAC, these approaches could help prioritize pathways such as XO–ROS signaling, EV-mediated communication, Th17/IL-17 activation, and endothelial NO-axis dysfunction. However, these mappings remain hypothesis-generating and should not be treated as clinically actionable. Prospective validation, cross-center calibration, and mechanistic perturbation studies are required before AI × multi-omics models can inform individualized intervention strategies (Figure 6).

## 7 Interventional strategies: mechanism-targeted approaches and evidence boundaries in UA-associated CAC

Before discussing specific interventions, it is important to define the current evidence boundary. Most UA-targeted studies have focused on serum UA control, renal outcomes, gout-related endpoints, or composite cardiovascular events. Few have directly evaluated CAC progression, CAC microstructure, or coronary calcification-specific trajectories. Therefore, mechanistic benefits observed in experimental models should not be directly extrapolated to reduced CAC progression or fewer cardiovascular events in DKD. The major translational gaps are summarized in Table 4.

**Table 4:** Clinical evidence boundaries and translational gaps of UA-targeted strategies in DKD-associated CAC.

Strategy	Main evidence type	CAC-specific endpoint	Cardiovascular endpoint	Main limitation	Appropriate interpretation
XO inhibitors	Animal/cell studies; small-to-moderate clinical studies	Limited; rarely used as primary endpoint	Inconsistent	Endpoint heterogeneity; insufficient CAC-focused trials	Exploratory or adjunctive strategy for XO–ROS-dominant phenotypes
Urate transporter inhibitors	Mechanistic studies; urate-lowering and safety studies	Largely lacking	Not established	Few studies assess CAC, EV biomarkers, or vascular microstructure	Hypothesis-generating strategy for renal urate deposition or EV-high phenotypes
Anti-inflammatory/antioxidant combinations	Preclinical evidence; small clinical cohorts	Mostly indirect	Unclear	Population heterogeneity; lack of mechanism-stratified trials	Candidate adjunctive strategy for high-inflammatory-burden subgroups
EV/radiomics-guided stratification	Methodological and conceptual evidence	Not prospectively validated	Not prospectively validated	No validated UA-specific CAC prediction model	Research framework for future validation
Early composite endpoints	Proposed based on imaging, EV, inflammasome, and metabolic biomarkers	Conceptual or early-stage	Not established	Temporal ordering and trigger thresholds remain undefined	Future trial design direction, not clinical guideline

### 7.1 XO inhibitors: mechanistic promise and clinical heterogeneity

XO is a biologically plausible therapeutic node because it links UA production, oxidative stress, endothelial dysfunction, and VSMC osteogenic activation (104). Animal and cell studies suggest that XO inhibition can reduce ROS burden, partially restore endothelial NO signaling, and attenuate osteogenic transcriptional programs in VSMCs (74). However, clinical evidence remains heterogeneous. Many studies have assessed renal function, serum UA control, gout-related outcomes, or composite cardiovascular endpoints rather than CAC progression as a primary endpoint (105). This endpoint mismatch limits direct translation from mechanistic improvement to CAC-specific clinical efficacy. Thus, XO inhibition is best positioned as an exploratory or adjunctive strategy for XO–ROS-dominant or NO-axis-dysregulated phenotypes, rather than a universal approach for preventing CAC progression in DKD (106).

### 7.2 UA transporter inhibitors: candidate strategies for renal UA load reduction

Urate transporter inhibitors targeting URAT1 or GLUT9 may reduce renal UA handling burden and local urate deposition, thereby indirectly weakening renal inflammatory signaling (107). Preclinical studies suggest that reduced renal UA load may be accompanied by lower mitochondrial stress and attenuated inflammasome activation (38).

However, most clinical studies of urate transporter inhibitors have focused on urate-lowering efficacy and safety rather than CAC progression, EV biomarkers, or coronary microstructural changes (108). Their role in DKD-associated CAC remains hypothesis-generating. Future studies should determine whether patients with renal urate deposition-dominant or EV-high phenotypes derive greater imaging or biomarker benefits from transporter-targeted strategies (95).

### 7.3 Anti-inflammatory and antioxidant combinations: candidate adjunctive approaches

Because UA-associated pathology may involve oxidative stress, inflammation, and vascular remodeling, combined anti-inflammatory and antioxidant strategies are biologically plausible (109). Animal and *in vitro* studies suggest that these approaches can reduce inflammatory signaling and osteogenic gene expression in vascular calcification-related models (110). Clinical evidence remains limited and is mainly derived from small cohorts or indirect biomarker analyses. Available population-level evidence suggests that imaging or biomarker benefits may be more detectable in subgroups with elevated inflammatory burden (111). Therefore, combined anti-inflammatory and antioxidant interventions should be described as candidate adjunctive strategies for selected high-inflammatory-burden phenotypes, not established first-line treatments for DKD-associated CAC.

### 7.4 Therapeutic windows and evidence boundaries of reversibility

The concept of a reversible therapeutic window is promising but not fully validated. Radiomics and translational studies suggest that texture roughness, low-attenuation regions, and edge morphology may reflect more active or plastic stages of calcification (97, 99, 112). Interventions targeting metabolic stress and cellular injury are more likely to be effective before calcification becomes structurally mature. In contrast, strong clinical evidence supporting reversal of established lamellar calcification remains limited (113).

**Figure 7:**
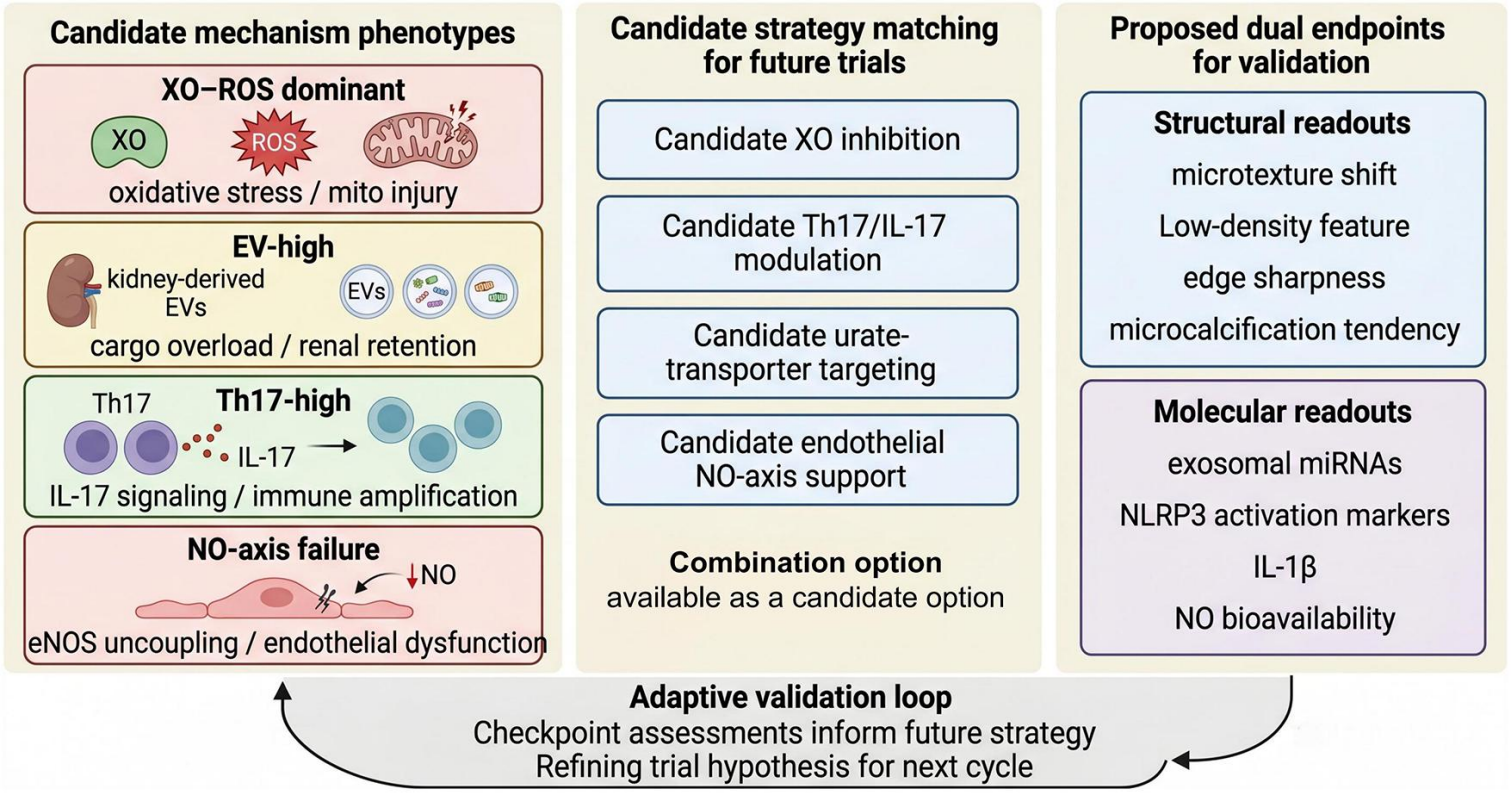
Proposed mechanism-based stratification and candidate strategy-matching framework for future validation studies in UA-associated CAC. The figure summarizes four candidate mechanistic phenotypes and corresponding exploratory intervention directions, together with proposed structural and molecular endpoints. This framework is intended to guide future mechanism-stratified trials rather than to serve as an established clinical treatment algorithm.

Overall, upstream interventions such as XO inhibition and urate transporter inhibition may attenuate disease-related pathways in selected mechanistic subgroups, while combination strategies may be more relevant in patients with high inflammatory burden or multi-pathway activation. However, therapeutic heterogeneity remains substantial, partly because of endpoint mismatch and insufficient mechanistic stratification (106). Future studies should prioritize early composite endpoints that combine CAC microstructural imaging, EV-associated inflammatory markers, and metabolic biomarkers. Stratified randomization, multicenter external validation, and transparent reporting standards will be essential. These proposals represent translational research directions rather than established clinical guidelines or universally applicable therapeutic pathways (Figure 7).

## 8 Outlook and future directions: evidence boundaries and validation priorities

This review proposes a UA-associated framework linking renal metabolic stress, immune propagation, vascular vulnerability, omics-based evidence layers, and candidate intervention strategies. However, many components remain hypothesis-generating. Future work should focus on causal validation, technical standardization, prospective clinical testing, and clearer definition of potentially reversible disease stages.

### 8.1 Mechanistic uncertainties requiring causal validation

A key unresolved question is how UA-associated metabolic stress develops into sustained inflammatory activation across renal and immune cell types. Although NLRP3 activation has been linked to hyperuricemia, its activation threshold, duration, and cell-type specificity remain unclear (40). Future studies should combine metabolic flux tracing, single-cell time-series profiling, and perturbation experiments to distinguish transient adaptive stress from sustained inflammatory remodeling (32, 114).

These approaches may help determine whether UA-associated pathways act as initiating triggers, amplifiers, or biomarkers of broader DKD-related injury.

### 8.2 Technical requirements for AI × multi-omics integration

For AI × multi-omics integration, reproducibility and interpretability are as important as model performance. Imaging models require standardized feature extraction, intensity normalization, spatial resampling, and robustness filtering. Omics datasets require batch correction and cross-platform harmonization (115, 116). External validation and transparent reporting should follow established principles such as TRIPOD- and CONSORT-AI-oriented reporting, model cards, and data descriptors (117, 118).

At this stage, AI × multi-omics integration should be considered a methodological platform for hypothesis generation and validation planning, not a clinically validated decision-support system.

### 8.3 Principles for mechanism-stratified prospective studies

Future studies should move beyond uniform urate-lowering strategies and adopt mechanism-stratified designs. Candidate stratification variables include serum UA dynamics, renal function, inflammatory biomarkers, EV-associated signatures, endothelial NO-axis markers, and CAC microstructural phenotypes. Standardized longitudinal CAC assessment and prespecified subgroup analyses are needed to determine whether UA-targeted approaches benefit specific biological phenotypes rather than the overall DKD population. Interaction testing should be planned in advance to evaluate whether treatment effects differ across XO–ROS-dominant, inflammation-dominant, EV-high, or NO-axis-dysregulated subgroups. This design would better connect mechanistic evidence with clinically meaningful CAC-related endpoints.

### 8.4 Candidate reversible windows and evidence boundaries

The reversible window of UA-associated CAC remains a research hypothesis rather than an established clinical stage. Current evidence suggests that early metabolic dysregulation, cellular stress, EV-associated signaling, and active microcalcification may represent more plastic stages than established lamellar calcification (119). Future studies should determine whether combined imaging and biomarker trajectories can identify these early stages before progression to low-reversibility calcification. Studies should also test whether intervention-related biomarker changes align temporally with CAC microstructural changes. Until such evidence is available, reversible-window models should be presented as research frameworks rather than clinical treatment algorithms.

**Figure 8:**
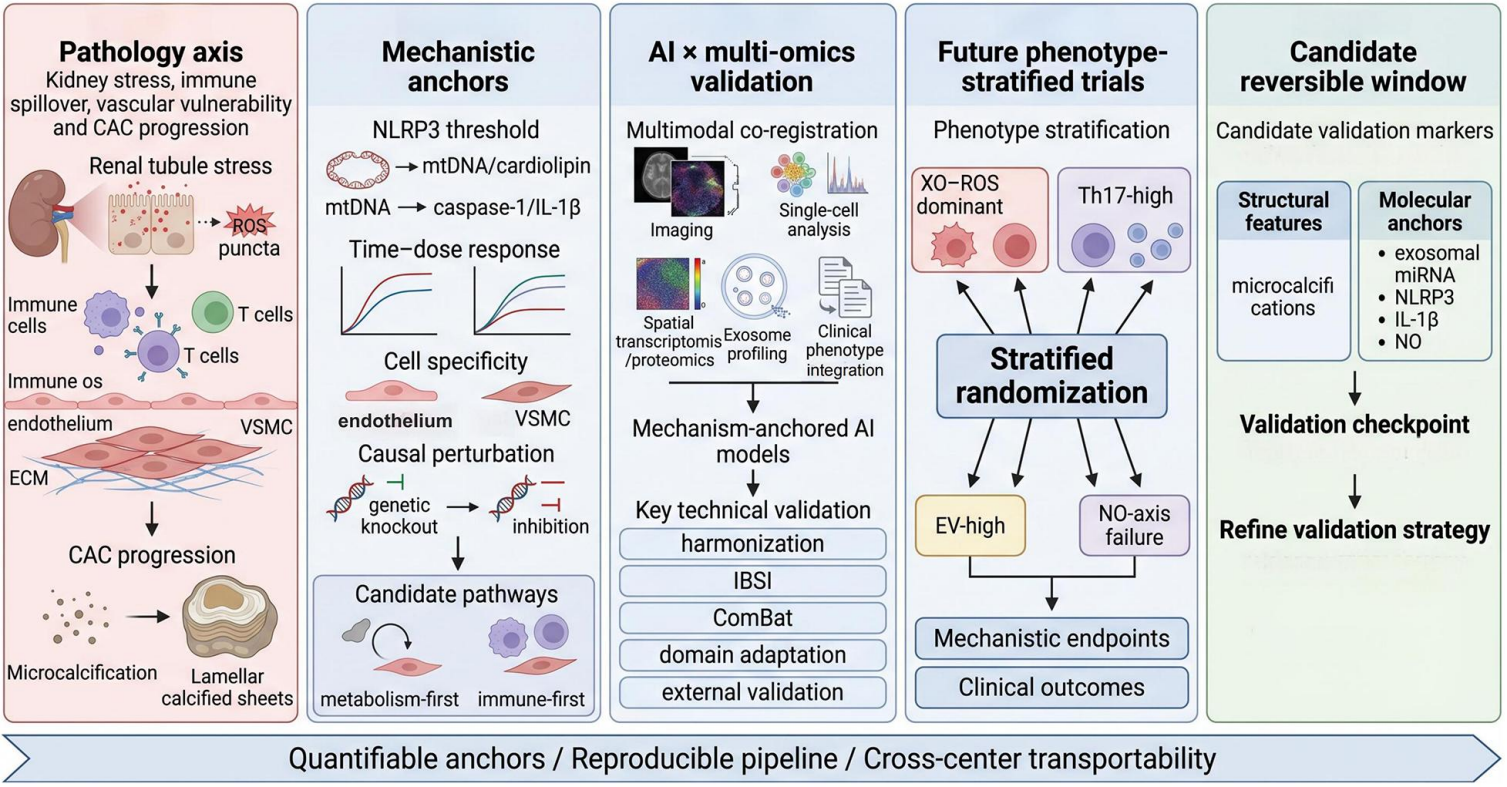
Conceptual translational framework linking mechanistic anchors, AI × multi-omics integration, phenotype-stratified trial design, and candidate reversible-window validation in UA-associated CAC. The framework highlights validation priorities, including quantifiable pathological nodes, reproducible AI–multi-omics pipelines, external validation, and cross-center transportability. It should be interpreted as a future research roadmap rather than a clinical decision algorithm.

Overall, this review proposes a three-stage translational pathway for UA-associated CAC: first, define quantifiable pathological nodes through mechanistic studies; second, map cellular, spatial, EV, imaging, and clinical phenotypes through interpretable AI × multi-omics integration; and third, test mechanism-stratified interventions in prospective randomized studies. Standardized data acquisition, transparent model development, external validation, and open reproducible practices will be essential for moving from mechanistic discovery toward individualized intervention strategies in multicenter settings (Figure 8).

## 9 Conclusion

The coexistence of DKD and CAC is associated with accelerated vascular calcification and poor cardiorenal outcomes. Current evidence suggests that UA is linked to renal metabolic stress, mitochondrial dysfunction, inflammasome activation, immune propagation, and calcification-prone vascular remodeling. However, these associations should be interpreted within the broader multifactorial context of DKD-associated CAC.

Single-cell, spatial, EV-based, radiomics, and AI-related approaches provide complementary tools for organizing existing evidence and generating testable hypotheses. These methods may help identify vulnerable renal and vascular cell states, candidate cross-organ communication routes, and imaging phenotypes related to rapid CAC progression. Nevertheless, most proposed UA–kidney–immune–vascular links remain inferential, and their causal directionality requires further validation.

Overall, UA may serve as a biologically informative marker and potential contributor within a broader cross-organ network connecting DKD and CAC. Future prospective studies are needed to clarify causality, CAC-specific clinical relevance, and the interventional value of UA-targeted strategies.
